# Supplementary material for: The impact of COVID-19 on allied health professions
Source: PLoS One. 2020 Oct 30;15(10):e0241328. doi: 10.1371/journal.pone.0241328 (PMC7598457; doi:10.1371/journal.pone.0241328)
Supplement: S1 File — This file includes the survey used in the current study. (PDF) [file pone.0241328.s001.pdf]

The Impact of the COVID-19 Pandemic on Non-Physician Healthcare Professions

---

Start of Block: Consent

Q33 **The Impact of COVID-19 on Non-Physician Healthcare Professions** You have been asked to participate in this study because you are a non-physician healthcare professional who may be impacted by the COVID-19 pandemic. The purpose of this study is to understand how the pandemic has affected you in terms of employment, your access to testing and personal protection equipment and the impact on your mental health during this time. Participation in this study will take 5-10 minutes of your time. There are no foreseeable risks or benefits to you for participating in this study except discomfort when answering some of the questions. You have the option to skip a question if you do not feel comfortable. There is no cost or payment to you. **You *will* remain anonymous and your answers are confidential. Please do not write your name anywhere in the survey.** If you have questions for one of the researchers conducting this study, you may contact Sandra Prentiss, Ph.D. at s.prentiss@med.miami.edu. If you have questions regarding your rights as a research participant, contact the University of Miami, Human Subject Research Office at (305)243-3195. Your participation in this research is voluntary, and you will not be penalized or lose benefits if you refuse to participate or decide to stop. *<clicking “yes” indicates your consent to participate>*

○ Yes (1)

○ No (2)

Skip To: End of Survey If The Impact of COVID-19 on Non-Physician Healthcare Professions You have been asked to participa... = No

End of Block: Consent

Start of Block: Demographics

Q1 What is your age?

- 18-24 years (1)
- 25-34 years (2)
- 35-44 years (3)
- 45-54 years (4)
- 55-64 years (5)
- 65 years or older (6)

Q2 Sex:

- Male (1)
- Female (2)
- Other (3)
- Prefer not to answer (4)

Q3 Household Size (Including yourself):

- 1-2 (1)
- 3-4 (2)
- 5-6 (3)
- 7 or more (4)

End of Block: Demographics

Start of Block: Employment

Q4 What is your current position?

- Faculty (1)
- Staff (2)
- Post-Doctoral Fellow (3)
- Graduate Student (4)
- Student (5)

Q5 What is your primary clinical setting?

- University- Hospital Setting (1)
- University- Non-Hospital Setting (2)
- Hospital Setting (3)
- VA Hospital (10)
- Nursing Home (4)
- Private Practice (5)
- Manufacturer (6)
- Rehabilitation Center (7)
- Retail Setting (8)
- School/Educational Setting (9)
- Other: (11) ____

Q7 What profession best describes you?

- ○ Audiologist (1)
- ○ Audiology Assistant (2)
- ○ Dental Hygienist (20)
- ○ Mental Health Counselor (4)
- ○ Occupational Therapist (6)
- ○ Occupational Therapy Assistant (7)
- ○ Optometrist (8)
- ○ Optometric Assistant (9)
- ○ Physical Therapist (10)
- ○ Physical Therapy Assistant (11)
- ○ Physician Assistant (12)
- ○ Psychologist (13)
- ○ Respiratory Therapist (14)
- ○ Respiratory Therapy Assistant (15)
- ○ Speech Language Pathologist (16)
- ○ Speech Language Pathology Assistant (17)
- ○ Social Worker (18)
- ○ Other: (19) ____

Q8 How long have you been working in your current position?

- ○ 0-5 years (1)
- ○ 6-10 years (2)
- ○ 11-15 years (3)
- ○ 16-20 years (4)
- ○ 21 years or more (5)

Q9 In which state do you currently work?

▼ Alabama (1) ... I do not reside in the United States (53)

Q11 Which patient population do you work with?

- ○ Adults (1)
- ○ Pediatrics (2)
- ○ Both adults and pediatrics (3)

End of Block: Employment

Start of Block: COVID-19 Response

Q12 What is the current status of your office or facility?

- ○ Open (1)
- ○ Open in a limited capacity (ie: Emergencies only) (2)
- ○ Closed with plans to re-open (3)
- ○ Closed with no plans to re-open (4)

Display This Question:

If What is the current status of your office or facility? = Open

Or What is the current status of your office or facility? = Open in a limited capacity (ie: Emergencies only)

Q13 If your office or facility is open or open in a limited capacity, are you: (select all that apply)

- ☐ Seeing patients on-site daily (1)
- ☐ Seeing emergent cases only (2)
- ☐ Seeing patients via Telehealth (3)
- ☐ Working from home (4)

Display This Question:

If If your office or facility is open or open in a limited capacity, are you: (select all that apply) = Working from home

Q13 What tasks are you completing from home? (select all that apply)

- ☐ Administrative activities (1)
- ☐ Publications (2)
- ☐ Updating clinical protocols (3)
- ☐ Academic research (4)

Display This Question:

If If your office or facility is open or open in a limited capacity, are you: (select all that apply) = Working from home

Q14 Please explain how productivity is measured during this time: (select all that apply)

- ☐ Routine check-in with supervisor (1)
- ☐ Time tracking (2)
- ☐ Project management tracker (ex: Microsoft Teams) (3)
- ☐ Other: (4) ____

Display This Question:

If What is the current status of your office or facility? = Closed with plans to re-open

Or What is the current status of your office or facility? = Closed with no plans to re-open

Q15 How long has your office been closed?

- ○ 1-6 days (1)
- ○ 1 week (2)
- ○ 2 weeks (3)
- ○ 3 weeks (4)
- ○ 4 weeks (5)
- ○ 5 weeks (6)
- ○ 6 weeks or more (7)

Display This Question:

If What is the current status of your office or facility? = Closed with plans to re-open

Or What is the current status of your office or facility? = Closed with no plans to re-open

Q35 Do you agree with your clinic's decision to close?

- Strongly agree (1)
- Agree (2)
- Disagree (3)
- Strongly disagree (4)

Display This Question:

If What is the current status of your office or facility? = Open

Or What is the current status of your office or facility? = Open in a limited capacity (ie: Emergencies only)

Q16 Do you agree with your clinic's decision to remain open?

- Strongly agree (1)
- Agree (2)
- Disagree (3)
- Strongly disagree (4)

Q17 What is your current employment status?

- Employed (1)
- Employed with salary reduction (2)
- Laid-Off (3)
- Furloughed (4)
- Forced to take Paid Time Off (PTO) with unpaid leave after my PTO runs out (5)
- Forced to take PTO and my PTO balance will go negative after my PTO runs out (6)

Display This Question:

If What is your current employment status? = Employed

Or What is your current employment status? = Employed with salary reduction

Q18 Are you continuing to see patients under your scope of practice or have you been reassigned to perform tasks outside of normal clinical duties?

- ○ I am continuing to see patients under my scope of practice (1)
- ○ I have been reassigned to perform tasks outside of my normal clinical duties. (2)

Display This Question:

If Are you continuing to see patients under your scope of practice or have you been reassigned to pe... = I have been reassigned to perform tasks outside of my normal clinical duties.

Q19 If you have been reassigned to perform tasks outside of your normal clinical duties, please select which tasks you have been assigned: (check all that apply)

- ▢ Taking temperatures (1)
- ▢ Screening patients (2)
- ▢ Scheduling patients (3)
- ▢ Triaging patients (4)
- ▢ Other: (5) ____

Q20 Please indicate how much you agree or disagree with the following statement: I feel stressed regarding the changes in my clinical practice due to the COVID-19 Pandemic.

- Strongly agree (1)
- Agree (2)
- Disagree (3)
- Strongly disagree (4)

Q21 Do you have access to any of the following mental health support? (check all that apply)

- Mental health counselor/psychologist (1)
- Webinar focused on mental health (2)
- Apps (ex: Headspace, Calm) (3)
- Support Group (4)
- Other (5)
- No, I do not have access to any of these supports (6)
- Unsure (7)

Display This Question:

If Do you have access to any of the following mental health support? (check all that apply) = Mental health counselor/psychologist

Or Do you have access to any of the following mental health support? (check all that apply) = Webinar focused on mental health

Or Do you have access to any of the following mental health support? (check all that apply) = Apps (ex: Headspace, Calm)

Or Do you have access to any of the following mental health support? (check all that apply) = Support Group

Or Do you have access to any of the following mental health support? (check all that apply) = Other

Q22 If yes, have you utilized any of the following services: (check all that apply)

- ☐ Mental health counselor/psychologist (1)
- ☐ Webinar focused on mental health (2)
- ☐ Apps (ex: Headspace, Calm) (3)
- ☐ Support Group (4)
- ☐ Other: (5) ____
- ☐ N/A (I have not utilized any of these services) (6)

Q23 How important is access to mental health services during this time?

- ○ Very important (1)
- ○ Important (2)
- ○ Not important (3)

Q24 Have you or your clinic implemented any restrictions or screenings for patients before they are seen? (Check all that apply)

- ▢ Verbal screening for symptoms (ex: Do you have a cough, have you traveled recently, etc.) (1)
- ▢ Physical screening for symptoms (ex: Taking temperatures) (2)
- ▢ Physical distance (ex: Hearing aid drop box, picking up devices from a patient's car) (3)
- ▢ Limit the patient to one companion (4)
- ▢ Limit the patient to no companions (5)
- ▢ Other: (6) ____
- ▢ No restrictions implemented (7)
- ▢ N/A (office is closed) (8)

Q25 Do you have access to personal protective equipment (PPE) at work?

- ○ Yes (1)
- ○ No (2)
- ○ N/A (3)

Display This Question:

If Do you have access to personal protective equipment (PPE) at work? = Yes

Q26 If yes, please check which types of PPE you have access to at work (select all that apply)

▢ N-95 Masks (1)

▢ Surgical Masks (2)

▢ Surgical Masks with Face Shield (3)

▢ Homemade Masks (4)

▢ Gloves (5)

▢ Goggles (6)

▢ Other: (7) ____

Q26 Do you believe that your profession is essential during the COVID-19 Pandemic?

◯ Yes (1)

◯ Yes, but in a limited capacity (2)

◯ No (3)

Q27 Do you have access to COVID-19 testing through your place of employment?

◯ Yes (1)

◯ No (2)

◯ Unsure (3)

Q28 Have you been tested for COVID-19 or COVID-19 Antibodies? (select all that apply)

- ☐ Yes, I have been tested for COVID-19 (1)
- ☐ Yes, I have been tested for COVID-19 Antibodies (2)
- ☐ No (3)

Display This Question:

If Have you been tested for COVID-19 or COVID-19 Antibodies? (select all that apply) = Yes, I have been tested for COVID-19

Q29 Was the test for COVID-19 positive or negative?

- ○ Positive (1)
- ○ Negative (2)
- ○ Unsure (I did not receive results or am waiting for results) (3)

Display This Question:

If Have you been tested for COVID-19 or COVID-19 Antibodies? (select all that apply) = Yes, I have been tested for COVID-19 Antibodies

Q34 Was the test for COVID-19 Antibodies positive or negative?

- ○ Positive (1)
- ○ Negative (2)
- ○ Unsure (I did not receive results or am waiting for results) (3)

End of Block: COVID-19 Response

Start of Block: COVID-19 Impact

Q30 How concerned are you about acquiring COVID-19 at work?

- Extremely concerned (1)
- Very concerned (2)
- Slightly concerned (3)
- Not at all concerned (4)
- N/A (I am not going to work) (5)

Q31 How concerned are you about acquiring COVID-19 outside of work? (ex: grocery store, pharmacy, from family members, outdoor exercise, etc.)

- Extremely concerned (1)
- Very concerned (2)
- Slightly concerned (3)
- Not at all concerned (4)

Q32 How concerned are you about transmitting COVID-19 to your family or to others?

- Extremely concerned (1)
- Very concerned (2)
- Slightly concerned (3)
- Not at all concerned (4)

End of Block: COVID-19 Impact
