# Supplementary material for: The impact of COVID-19 on allied health professions
Source: PLoS One. 2020 Oct 30;15(10):e0241328. doi: 10.1371/journal.pone.0241328 (PMC7598457; doi:10.1371/journal.pone.0241328)
Supplement: Attachment — Submitted filename: Response to Reviewers.docx [file pone.0241328.s002.docx]

Sandra Prentiss, PhD

Associate Professor

University of Miami Miller School of Medicine

Department of Otolaryngology

Rosemary Frey
Academic Editor

*PLOS ONE*

July 24, 2020

Dear Ms. Frey,

We would like to thank you and the reviewers for your thoughtful review of our manuscript entitled, “*The Impact of COVID-19 on Allied Health Professions*”. We have carefully reviewed and addressed each comment and suggestion provided to us. Attached to this letter, you will find a table with all of the editor and reviewer comments and how each was addressed. As requested, you will find an uploaded version of the manuscript with tracked changes along with a clean version.

Thank you again for your continued interest in our manuscript and the opportunity to revise it. The reviewer comments and suggestions have greatly strengthened the study and we thank you for the opportunity to publish these results in PLOS ONE.

We look forward to hearing from you soon.

Sincerely,

Sandra Prentiss, PhD

Associate Professor

University of Miami Miller School of Medicine

Department of Otolaryngology

| **reviewer Comments**  **(Editor)** | **Response** | **location of change** |
| --- | --- | --- |
| 1. Please also strengthen your discussion section supported by citations to empirical research | Thank you for your feedback. We have strengthened our discussion by including a more comprehensive literature search and citations. | NA |
| 2. Please address the methodological issues raised by both reviewers | We have addressed the methodological issues raised by the reviewers and discussed in our limitations section. | NA |
| 3. Please include additional information regarding the survey or questionnaire used in the study and ensure that you have provided sufficient details that others could replicate the analyses.  For instance, if you developed a questionnaire as part of this study and it is not under a copyright more restrictive than CC-BY, please include a copy, in both the original language and English, as Supporting Information. Moreover, please include more details on how the questionnaire was pre-tested, and whether it was validated. | We have added more detail regarding our survey development and have also uploaded the survey as supporting information.  The survey was developed by the research team using published research available at the time of this study. It was not validated prior to use in this survey study. | Lines 166-169 |
| 4. Please ensure that your manuscript meets PLOS ONE's style requirements, including those for file naming. | We have reviewed the manuscript to ensure that it is consistent with the PLOS ONE style requirements and guidelines. | Throughout document  Files have been saved according to PLOS ONE’s syle requirements. |
| 5. In your Methods section, please provide additional information about the participant recruitment method and the demographic details of your participants.  Please ensure you have provided sufficient details to replicate the analyses such as:  a) the recruitment date range (month and year),  b) a description of any inclusion/exclusion criteria that were applied to participant recruitment,  c) a table of relevant demographic details,  d) a statement as to whether your sample can be considered representative of a larger population,  e) a description of how participants were recruited. | We have specified that the survey was open for seven days (4/16/2020 through 4/23/2020; see line 151). Exclusion criteria was also made clearer in the methods section. Demographic information of the survey participants is included in Table 1 and a limitations section has been added, which includes a generalizability statement. Lastly, we added more detailed information regarding participant recruitment. | Lines 128-136; 161; 432-437 |
| 6. We note that Figure 1 in your submission contains map images which may be copyrighted | We have reviewed the copyright for the map utilized in the manuscript. According to <https://mapchart.net/feedback.html> the image may be use for private or commercial use/publications freely as long as mapchart.net is referenced in the work. We have referenced mapchart.net in Figure 1. Please see screenshot from the website at the bottom of this document. | The figure has the copyright information on the bottom left. |
| 7. PLOS requires an ORCID iD for the corresponding author in Editorial Manager | The ORCID ID for Dr. Prentiss is 0000-0001-7195-7254 | The corresponding author’s ORCID ID has been linked with PLOS ONE |
| **reviewer Comments**  **(Reviewer 1)** | **Response** | **location of change** |
| 1. There are a few typos throughout that need correcting e.g. abstract: page 2 line 37 'of' is missing from the sentence "...impacted with regards to employment in the form pay reductions...." | We have reviewed the entire manuscript and have corrected any typos and suggested revisions. | Throughout |
| 2. Please ensure a copy of the survey is available in supplementary information. | We have included the survey as supplementary information. | Included and submitted as S1_File.pdf |
| 3. Methods - how many surveys were sent out in total? What was the response rate? | Surveys were distributed via professional organization email listservs and social media; therefore, the response rate is unable to be calculated. | NA |
| 4. Please provide a more comprehensive rationale for excluding EMT's given that they are considered allied health professionals? | We have added a more detailed justification, including literature on our decision to exclude EMTs. As noted in the literature, EMTs are at a higher risk of infection and stress even in the absence of a pandemic (Orellana et al., 2016; Bentley et al., 2013). Thus, there responses would have likely differed significantly than other allied healthcare professionals. We have added this as a limitation and also recommended that future research examine allied health professionals who are at higher risk, such as EMTs. | Lines 130-134 |
| 5. Given the variation in COVID19 cases. transmission and public health response by region, how might this have impacted on the regional response rate? | We appreciate the reviewer’s thoughtful feedback. We understand the concern about the transmission rates by region; however, this survey study was completed April 16-23 during the same time that the Center for Medicare and Medicaid Services recommended limiting all non-essential medical services. In addition, although we were able to capture respondents across all regions in the US, we do not have a large enough sample size in each region to examine differences among them. | NA |
| 6. On page 8 line 157 the authors state that they developed the survey based on literature - what literature specifically? Please include references to the literature used. What clinical experience did the research team have and how did this inform the development of the survey? | The research team is made up of clinical audiologists, psychologists, and researchers. Psychologists are trained in social/behavioral research, as well as instrument development. Dr. Cejas has specifically published on instrument development and is a NIH researcher, who has development measures.  As requested, we have also identified the research that was reviewed and used to develop the survey used in this study. We have ensured that the citations are included (6-9, 16-17). | Lines 166-169 |
| 7. Throughout the discussion there are large pieces of text that do not have references. For example page 15 lines 296-298 "The CDC and WHO provided guidance for frontline healthcare workers regarding PPE, primarily focusing on physicians and nurses". Please reference the guidelines.  The statement that PPE was initially being saved for those working with positive COVID-19 patients. Can this be referenced? How was this information found?  Page 15 lines 313-315 "Emergency legislation was passed which allowed for flexibility..." Please reference the legislation  Uncertainty regarding reimbursement was referred to - can this be referenced with the information source?  The discussion would benefit from references to government or local guidelines that support some of the arguments used. | We have thoroughly reviewed the discussion and added any necessary references. | Throughout discussion |
| **reviewer Comments**  **(Reviewer 2)** | **Response** | **location of change** |
| 1. This is an interesting paper but would be strengthened if the authors reported findings against the CHERRIES checklist (Equator network) https://www.ncbi.nlm.nih.gov/pmc/articles/PMC1550605/?report=reader#!po=50.0000 | Thank you for your comment and the reference. The survey indeed followed that of the CHERRIES checklist in design, distribution, time frame for data collection and statistical analysis. This is explained in the methods section. We could not calculate a response rate as the surveys were listed on websites and social media. Therefore, we do not have access to the number of people who had access to the survey and did not complete. | NA |
| 2. There is no real limitations section and some of the claims in conclusion and discussion need modifying | We have added a limitations section as requested. We have also reviewed our conclusions to ensure that they are all supported by the data. | Lines 417-437; Throughout discussion |
