# Supplementary material for: The impact of COVID-19 on allied health professions
Source: PLoS One. 2020 Oct 30;15(10):e0241328. doi: 10.1371/journal.pone.0241328 (PMC7598457; doi:10.1371/journal.pone.0241328)
Supplement: Attachment — Submitted filename: Response to Reviewers.docx [file pone.0241328.s003.docx]

Sandra Prentiss, PhD

Associate Professor

University of Miami Miller School of Medicine

Department of Otolaryngology

Rosemary Frey
Academic Editor

*PLOS ONE*

September 9, 2020

Dear Ms. Frey,

We would like to thank you and the reviewers for your thoughtful review of our manuscript entitled, “*The Impact of COVID-19 on Allied Health Professions*”. We have carefully reviewed and addressed the minor revisions suggested by the reviewers. Specifically, we included a statement regarding the use of the CHERRIES checklist within the text and as a reference. As requested, you will find an uploaded version of the manuscript with tracked changes along with a clean version.

Thank you again for your continued interest in our manuscript and the opportunity to revise it. The reviewer comments and suggestions have been valuable and we thank you for the opportunity to publish these results in PLOS ONE.

We look forward to hearing from you soon.

Sincerely,

Sandra Prentiss, PhD

Associate Professor

University of Miami Miller School of Medicine

Department of Otolaryngology
